# Transition Metal Complexes and Radical Anion Salts of 1,10-Phenanthroline Derivatives Annulated with a 1,2,5-Tiadiazole and 1,2,5-Tiadiazole 1,1-Dioxide Moiety: Multidimensional Crystal Structures and Various Magnetic Properties

**DOI:** 10.3390/molecules19010609

**Published:** 2014-01-07

**Authors:** Yoshiaki Shuku, Kunio Awaga

**Affiliations:** Department of Chemistry & Research Center for Materials Science, Nagoya University, Furo-cho, Chikusa, Nagoya 464-8602, Japan; E-Mail: shiyuku.yoshiaki@d.mbox.nagoya-u.ac.jp

**Keywords:** spin-crossover complexes, intermolecular interactions, electron acceptors, radical anion salts

## Abstract

Advances in the molecular variety and the elucidation of the physical properties of 1,10-phenanthroline annulated with 1,2,5-thiadiazole and 1,2,5-thiadiazole 1,1-dioxide moieties have been achieved, and are described herein. A 1,2,5-thiadiazole compound, [1,2,5]thiadiazolo[3,4-*f*][1,10]phenanthroline (tdap), was used as a ligand to create multidimensional network structures based on S•••S and S•••N intermolecular interactions. A 1,2,5-thiadiazole 1,1-dioxide compound, [1,2,5] thiadiazolo[3,4-*f*][1,10]phenanthroline, 1,1-dioxide (tdapO_2_), was designed to create a stable radical anion, as well as good network structures. Single crystal X-ray structure analyses revealed that transition metal complexes of tdap, and radical anion salts of tdapO_2_ formed multidimensional network structures, as expected. Two kinds of tdap iron complexes, namely [Fe(tdap)_2_(NCS)_2_] and [Fe(tdap)_2_(NCS)_2_]•MeCN exhibited spin crossover transitions, and their transition temperatures showed a difference of 150 K, despite their similar molecular structures. Magnetic measurements for the tdapO_2_ radical anion salts revealed that the magnetic coupling constants between neighboring radical species vary from strongly antiferromagnetic (*J* = −320 K) to ferromagnetic (*J* = 24 K), reflecting the differences in their π overlap motifs.

## 1. Introduction

The electrical and magnetic properties of molecular crystals have been studied extensively, and various molecule-based conductors [1,2,3,4,5,6,7], superconductors [6,8,9,10,11,12,13,14], and magnetic materials [15,16,17,18,19,20,21,22,23,24,25,26,27] have been synthesized to date. The research has been characterized by the enhancement of dimensionality in intermolecular interactions. As a result of the major efforts to enhance the dimensionality of intermolecular interactions, heterocyclic sulfur nitrogen (SN) compounds, which consist of nitrogen atoms and sulfur atoms, have arisen, and their solid-state properties have been investigated in relation to their crystal structures. The heterocyclic chalcogen-nitrogen compounds have attracted much attention due to their potential to form a multidimensional network structure in their crystals, which involve X•••N and X•••X (X = S, Se, Te) contacts and π-overlaps. Furthermore, some of these compounds are known to be radical species without any protecting groups. These features are believed to bring about the unique chemical and physical properties of these materials, such as the high-*T*_c_ magnetic orderings, high conductivities and drastic structural phase transitions (see [28] and references therein). Although the intermolecular interactions are weaker than those of other heterocyclic chalcogen-nitrogen compounds, such as SeN and TeN compounds, various structures of heterocyclic SN compounds have been reported due to the convenience of their synthesis [28,29,30,31,32,33].

**Scheme 1:**
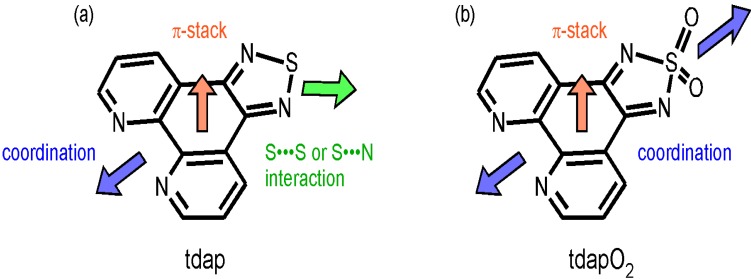
Molecular structures of tdap (**a**) and tdapO_2_ (**b**).

The synthesis of a closed-shell SN compound, [1,2,5]thiadiazolo[3,4-*f*][1,10]phenanthroline (tdap, Scheme 1a), was reported in 2006 [34]. Using this compound as a ligand, the intermolecular interactions and electron spins which are the remarkable features of SN radicals can be separated into the 1,2,5-thiadiazole moieties and the transition metal ions, respectively. By this method, the stability can be greatly improved, whilst simultaneously maintaining the open shell structure. In this study, a 1,2,5-thiadiazole moiety is used to introduce the intermolecular interaction to transition metal ions which have a large quantum spin number.

While the 1,2,5-thiadiazole compounds have been widely investigated in the field of solid state physics, research into their related 1,2,5-thiadiazole 1,1-dioxide compounds has been limited to solution electrochemistry, despite their unique electronic structures. Past studies of these compounds have revealed that they tend to be good electron acceptors [35]. The solution electron paramagnetic resonance (EPR) spectra of 1e reduced phenanthro[9,10-c]-1,2,5-thiadiazole 1,1-dioxide suggests the formation of a stable radical anion whose unpaired electron is delocalized over the molecule [36]. Radical anions are suitable building blocks for molecule magnets, when combined with appropriate transition metal ions. However, possibly due to the instability of the reduced species, the number of examples of radical anions is very limited. Some of the most successful radical anions are TCNE^−^ [25,37,38], TCNQ^−^ [37,39,40,41], DCNQI^−^ (DCNQI: *N*,*N*’-dicyanoquinonediimine) [42] and semiquinones [43,44,45]. Most notably, V•(TCNE)*x*•*y*CH_2_Cl_2_ (*x* ~ 2, *y* ~ 1/2) has been shown to exhibit ferrimagnetism, even at room temperature (*T*_N_ > 350 K) [46]. These radical anion complexes suggest the importance of stable electron acceptor molecules for molecule-based magnets and conductors. Recently, radical anion salts of [1,2,5]thiadiazolo[3,4-*f*][1,10]phenanthroline 1,1-dioxide (tdapO_2_, Scheme 1b) were reported and shown to be stable under ambient conditions and to have multidimensional network crystal structures [47,48].

In the present paper, we describe the crystal structures and spin-crossover transitions of tdap transition metal complexes, and the crystal structures and magnetic properties of radical anion salts of tdapO_2_.

## 2. Transition Metal Complexes of [1,2,5]thiadiazolo[3,4-*f*][1,10]phenanthroline

The synthesis of [1,2,5]thiadiazolo[3,4-*f*][1,10]phenanthroline (tdap, Scheme 1a) was reported in 2006 by Conte *et al.* [34]. They reported the luminescent properties of the Eu^III^ complex with the ligand in 2008 [49], and the photoluminescence and electroluminescence of the Tb^III^ complex in 2011 [50]. In addition, they reported the photonuclease activity of the Fe^II^ complex in 2010 [51]. In these papers, the crystal structures of these complexes were also reported. Thus there was no intermolecular interaction found, and the properties were discussed as single molecule features. Despite this report, we expected that the thiadiazole moiety of this ligand would have potential to form intermolecular interactions and would be a good building block for molecule-based magnets. In this chapter, we describe the crystal structures and intermolecular interactions of first-row transition metal complexes of tdap. Among them, the crystal structures and magnetic properties of two spin crossover complexes are discussed with emphasis on the intermolecular interactions and molecular structures. In this chapter, 10 kinds of novel tdap complexes ([Fe(tdap)_2_(NCS)_2_]•MeCN, [Co(tdap)_2_(NCS)_2_], [Mn(tdap)_2_(NCS)_2_]•MeCN, [Co(tdap)_2_(NCS)_2_]•MeCN, [Ni(tdap)_2_(NCS)_2_]•MeCN, [Cu(tdap)_2_(NCS)_2_]•MeCN, [Zn(tdap)_2_(NCS)_2_]•MeCN, [Mn(tdap)_2_Cl_2_], [Cu(tdap)_2_(NCS)_2_], [Cu(tdap)_2_(NCS)_4_]) are included.

### 2.1. [1,2,5]thiadiazolo[3,4-f][1,10]phenanthroline (tdap)

**Figure 1:**
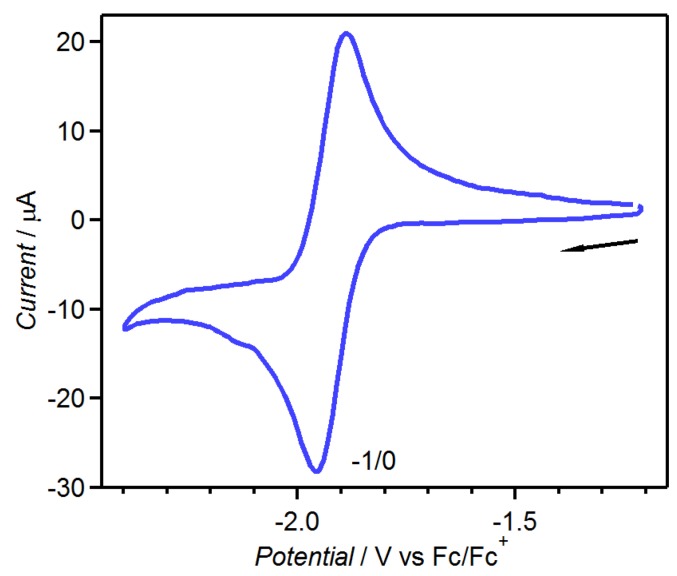
CV of 1 mM tdap in MeCN solution of 100 mM TBAClO_4_. Adapted with permission from [48]. Copyright 2013 American Chemical Society.

The electrochemical measurement of tdap in dimethyl sulfoxide (DMSO) has been reported, and revealed a redox peak in the reduction range (*E*_1/2_ = −1.46 V *vs.* a normal hydrogen electrode (NHE)) [51]. To compare the acceptor ability to the related compound tdapO_2_ described in a later section, the electrochemical measurement in acetonitrile (MeCN) was carried out (*E*_1/2_ = −1.93 V *vs.* Fc/Fc^+^ Figure 1, where Fc is ferrocene). These results suggest that tdap has poor acceptor ability.

### 2.2. Synthesis of Transition Metal Complexes of tdap

Single crystals of transition metal complexes of tdap were obtained by the slow diffusion method using an H-shaped glass cell purged with N_2_ gas [52]. Crystal solvent-free transition metal complexes [Fe(tdap)_2_(NCS)_2_] (black blocks) [53] and [Co(tdap)_2_(NCS)_2_] (orange needles) were obtained by the slow diffusion between an EtOH solution of MSO_4_•7H_2_O (M = Fe, M = Co) and KNCS, and a CH_2_Cl_2_ solution of tdap. Black prism crystals of [Fe(tdap)_2_(NCS)_2_]•CH_2_Cl_2_ and red platelet crystals of tris tdap complex [Fe(tdap)_3_]•(NCS)_2_ were obtained as a mixture with the crystals of [Fe(tdap)_2_(NCS)_2_], which were separated by viewing under a microscope [53]. Yellow platelet crystals of [Mn(tdap)_2_Cl_2_] were obtained in a similar manner using an EtOH solution of MnCl_2_•4H_2_O as a metal source.

Using a mixture of sulfonate salt of transition metal ion (MnSO_4_•5H_2_O, FeSO_4_•7H_2_O, CoSO_4_•7H_2_O, NiSO_4_•6H_2_O, CuSO_4_•5H_2_O or ZnSO_4_•7H_2_O) and potassium thiocyanate as metal sources and MeCN as a solvent, transition metal complexes of [M(tdap)_2_(NCS)_2_]•MeCN (M = Mn, Fe, Co, Ni, Cu, Zn), [Cu_2_(tdap)_2_(NCS)_2_] and [Cu_2_(tdap)_2_(NCS)_4_] were obtained. Green block crystals of [Cu(tdap)_2_(NCS)_2_]•MeCN, brown needle-shaped crystals of [Cu_2_(tdap)_2_(NCS)_2_] and green prismatic crystals of [Cu_2_(tdap)_2_(NCS)_4_] were obtained simultaneously and separated by viewing under a microscope. Crystallographic parameters for novel crystal structures are shown in the Supplementary Materials, Table S1.

### 2.3. Spin-Crossover Complexes of tdap

The behavior of SCO transitions is known to be greatly affected by intermolecular interactions. The magnetic properties and crystal structures of two SCO complexes of tdap are discussed in this study. The non-solvated [Fe(tdap)_2_(NCS)_2_] is reported to exhibit a SCO transition at 320 K [53]. The crystals of the solvated [Fe(tdap)_2_(NCS)_2_]•MeCN have been successfully obtained, and reveal a SCO transition at 210 K. The complex [Fe(tdap)_2_(NCS)_2_], without crystal solvent, exhibits effective S•••S intermolecular interactions. In contrast, [Fe(tdap)_2_(NCS)_2_], with a crystal solvent of MeCN, does not show effective intermolecular interactions. Hereafter, SCO transitions of [Fe(tdap)_2_(NCS)_2_]•MeCN and [Fe(tdap)_2_(NCS)_2_] are discussed from the view point of intermolecular interactions, by comparing them with the SCO transitions of [Fe(phen)_2_(NCS)_2_] (phen: 1,10-phenanthroline) [54].

#### 2.3.1. [Fe(tdap)_2_(NCS)_2_]•MeCN

**Figure 2:**
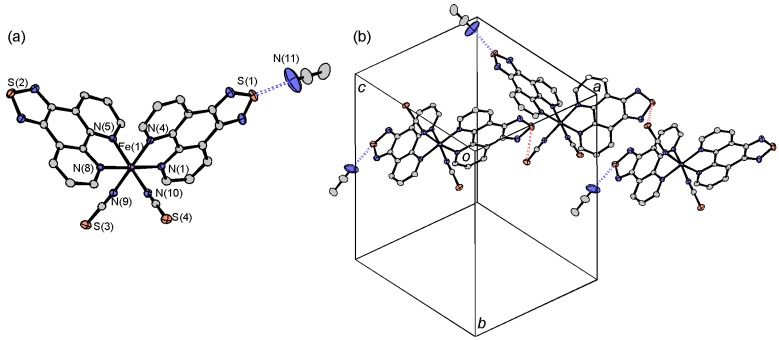
Molecular structure (**a**) and crystal packing (**b**) of [Fe(tdap)_2_(NCS)_2_]•MeCN.

The molecular structure of [Fe(tdap)_2_(NCS)_2_]•MeCN is shown in Figure 2a. The Fe^II^ ion is surrounded by six nitrogen atoms belonging to two isothiocyanate groups in *cis* positions, and two tdap ligands. Therefore, the molecule is chiral and the unit cell contains two right-handed and two left-handed enantiomers. The Fe-N bond lengths are typical for those of Fe^II^ SCO complexes both in the HS state (2.1838(15)–2.2244(15) Å for Fe-N_tdap_, and 2.0715(19) and 2.0953(15) Å for Fe-NCS) and LS state (1.9754(16)–1.9815(16) Å for Fe-N_tdap_, and 1.9538(17) and 1.9608(16) Å for Fe-NCS) as shown in Table 1. The transition was accompanied by an expansion of the Fe-N distances in the same manner as for previously reported SCO complexes [55].

**Table 1:** Selected bond lengths/Å of [Fe(tdap)_2_(NCS)_2_]•MeCN and [Fe(tdap)_2_(NCS)_2_].

	[Fe(tdap)_2_(NCS)_2_]•MeCN		[Fe(tdap)_2_(NCS)_2_]	
	320 K	120 K	400 K	173 K
Fe-N_tdap_	2.1838(15)	1.9786(17)	2.183(4)	1.966(3)
	2.2212(14)	1.9808(14)	2.196(4)	1.969(3)
	2.1843(15)	1.9815(16)	2.197(4)	1.978(2)
	2.2244(15)	1.9754(16)	2.233(4)	1.978(3)
Fe-NCS	2.0715(19)	1.9538(17)	2.092(6)	1.940(3)
	2.0953(17)	1.9608(16)	2.112(7)	1.948(3)
N-CS	1.157(2)	1.164(2)	1.117(9)	1.159(5)
	1.153(2)	1.161(2)	1.055(11)	1.159(5)

Both in the HS and LS states, the space groups were *P*2_1_/*n*, and there was a 5.5% difference in unit cell volume between the two states. This change results not only from the thermal expansion of the crystal lattice, but also from the expansion of the Fe-N distances upon the SCO transition. The crystal structure of [Fe(tdap)_2_(NCS)_2_]•MeCN, measured at 120 K, is shown in Figure 2b. In the LS state, the molecular packing exhibits S(1)•••N(11) contacts (3.017(4) Å: blue broken lines in Figure 2b), which are shorter than the sum of van der Waals (VDW) radii (3.35 Å), and weak S(2)•••S(4) (3.5911(7) Å: red broken lines in Figure 2b) contacts, which are almost the same as the sum of VDW radii (3.60 Å). Hereafter, the VDW radii given by Bondi were used (C: 1.75 Å; Cl: 1.75 Å; N: 1.55 Å; O: 1.50 Å; S: 1.80 Å) [56]. This weak S(2)•••S(4) contact forms a one-dimensional (1D) structure for the [Fe(tdap)_2_(NCS)_2_] complexes, while the S•••N short contacts quench the intermolecular interactions between complexes. After the transition to the HS state, the S(2)•••S(4) contacts show a 2.5% expansion, while the S(1)•••N(11) distance shows a 2.4% reduction. As a result, the S(2)•••S(4) distances elongated to 3.6805(8) Å, which is longer than the sum of the VDW radii, and the complexes showed less interaction in the HS state.

**Figure 3:**
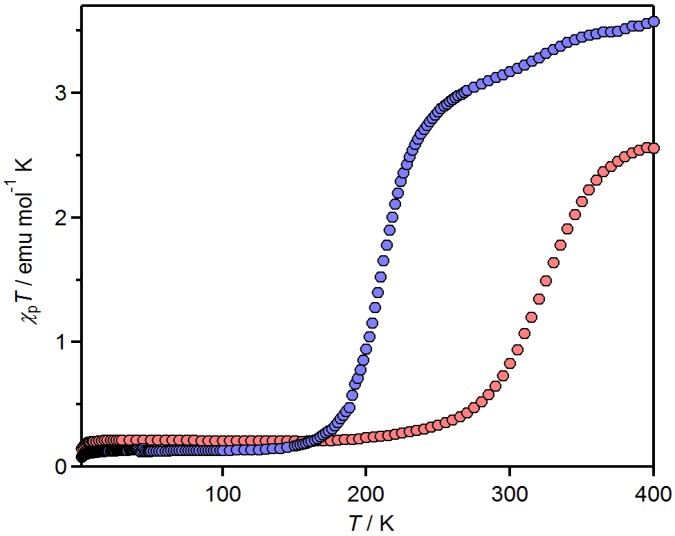
Temperature dependence of *χ*_p_*T* of [Fe(tdap)_2_(NCS)_2_]•MeCN (blue) and [Fe(tdap)_2_(NCS)_2_] (red) [53]. Adapted by permission of The Royal Society of Chemistry).

Magnetic susceptibility measurements of [Fe(tdap)_2_(NCS)_2_]•MeCN were performed under 0.5 T, in the temperature range of 2–400 K. Both the heating and cooling processes were performed to confirm the non-existence of structural change due to the evaporation of crystal solvent. The fact that the same profiles were observed in both processes suggests that no evaporation occurred even at 400 K. The paramagnetic susceptibility (*χ*_p_) of the complex was calculated with a diamagnetic susceptibility (*χ*_dia_) that was obtained by assuming that the temperature (*T*) dependence of *χ*_p_ followed the Curie law at low temperature. The temperature dependence of *χ*_p_ of [Fe(tdap)_2_(NCS)_2_]•MeCN is shown in Figure 3. Since there is no observable thermal hysteresis in the SCO transition, only the heating process is shown. At low temperature, below 120 K, the value of *χ*_p_*T* is nearly constant (0.13 emu mol^−1^ K), though the spin state is expected to be *S* = 0. This is probably partially due to the presence of the HS state and/or contamination of the paramagnetic complex. Above 150 K, *χ*_p_*T* shows a gradual increase, and reaches 3.57 emu mol^−1^ K at 400 K. This value is larger than the theoretical spin-only value for a ^5^*T*_2g_ ground state (3.0 emu mol^−1^ K with *g* = 2, where *g* is a g-factor), indicating spin-orbital coupling.

#### 2.3.2. [Fe(tdap)_2_(NCS)_2_]

**Figure 4:**
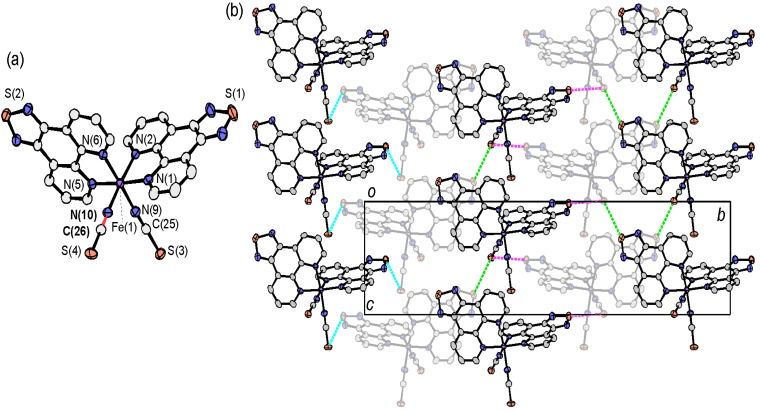
Three-dimensional network structure of solvent-free [Fe(tdap)_2_(NCS)_2_] at 173 K [53]. Adapted by permission of The Royal Society of Chemistry.

The solvent-free [Fe(tdap)_2_(NCS)_2_] was crystallized in the *Pna*2_1_ space group. The molecular structure and selected bond lengths of solvent-free [Fe(tdap)_2_(NCS)_2_] are shown in Figure 4a and Table 1, respectively. In the LS state, the Fe-N(tdap) distances are in the 1.966(3)–1.978(3) Å range and the Fe-NCS distances are 1.940(3) and 1.948(3) Å. In the HS state, Fe-N distances of [Fe(tdap)_2_(NCS)_2_] are 2.183(4)–2.197(4) Å (tdap), and 2.092(6) and 2.112(7) Å (NCS). In both states, bond distances are comparable for those of common SCO complexes in the LS state and HS state, respectively.

Figure 4b shows the crystal structure of [Fe(tdap)_2_(NCS)_2_] at 173 K, in which the complexes formed three kinds of S•••S short contacts between the thiadiazole rings and the NCS ligands (light blue lines: S(1)•••S(3) 3.3892(16) Å; pink lines: S(1)•••S(4) 3.3640(14) Å; light green lines: S(2)•••S(4) 3.5415(17) Å). These short S•••S contacts form a 3D network. This is probably attributable to the partial positive charge at the heterocyclic sulfur atom and the partial negative charge associated with the formally anionic NCS ligand.

After the transition from the LS to the HS state, the same space group, *Pna*2_1_, is retained, while the unit cell volume is increased by 7.4%. This change results from thermal expansion of the crystal lattice, and expansion of the Fe-N distances upon the SCO transition. The intermolecular interactions between complexes are also retained with slight elongations (light blue lines: S(1)•••S(3) 3.507(3) Å; pink lines: S(1)•••S(4) 3.467(2) Å; light green lines: S(2)•••S(4) 3.564(3) Å). The Fe-N bond lengths in [Fe(tdap)_2_(NCS)_2_] at 400 K are typical for an Fe^II^ HS species.

The comparison of the crystal structures in the HS state and LS state reveals several characteristic differences. It is notable that the N(10)-C(26) distance (for the NCS ligand) in the HS state is extremely short (see Figure 4a), presumably due to chemical pressure caused by the intermolecular contacts. The N-C(S) bond lengths at 400 K (1.117(9) Å for N(9)-C(25) and 1.055(11) Å for N(10)-C(26)) were 3.6% and 9.0% shorter than those at 173 K (1.159(5) Å for N(9)-C(25) and N(10)-C(26)), while [Fe(phen)_2_(NCS)_2_] [54] and [Fe(tdap)_2_(NCS)_2_]•MeCN exhibit a slight increase of 1.6% and a decrease of 0.6% in the N-C(S) distances, respectively. A comparison between the molecular structures of the solvated and non-solvated forms indicates that the large shrinkage in the latter would be caused by its characteristically short S•••S contacts. It is hard to clearly rationalize this difference, but the expansion of the FeN6 core could be balanced by such shrinkages in the non-solvated [Fe(tdap)_2_(NCS)_2_].

Magnetic susceptibility measurements of [Fe(tdap)_2_(NCS)_2_] were performed under 0.5 T, in the temperature range of 2–400 K. The *χ*_p_ of the complex was calculated with a *χ*_dia_ that was obtained by assuming that the temperature dependence of *χ*_p_ followed the Curie law. The temperature dependence of *χ*_p_ for [Fe(tdap)_2_(NCS)_2_] is shown in Figure 3. Below 150 K, the value of *χ*_p_*T* is nearly constant (0.21 emu mol^−1^ K), which is probably extrinsic. Above 180 K, *χ*_p_*T* shows a gradual increase, and reaches 2.56 emu mol^−1^ K at 400 K. This value is smaller than the theoretical spin-only value for a ^5^T_2g_ ground state (3.0 emu mol^−1^ K with *g* = 2), indicating an incomplete transition. It is notable that there is no thermal hysteresis in the SCO transition in [Fe(tdap)_2_(NCS)_2_], despite the intermolecular contacts in this compound. The unsubstituted 1,10-phenanthroline complex [Fe(phen)_2_(NCS)_2_] is known to exhibit a steep SCO transition at 177 K without hysteresis [54]. Since the FeN6 geometry in [Fe(tdap)_2_(NCS)_2_] is very similar to that in [Fe(phen)_2_(NCS)_2_], the intermolecular contacts in [Fe(tdap)_2_(NCS)_2_] are considered to bring about a high temperature shift of more than 100 K in the SCO transition. As described above, the intermolecular interactions of [Fe(tdap)_2_(NCS)_2_] disturb the molecular expansion of the SCO transition. As a result, the expanded structure in the HS state is destabilized, and the LS state is maintained to a high temperature.

**Figure 5:**
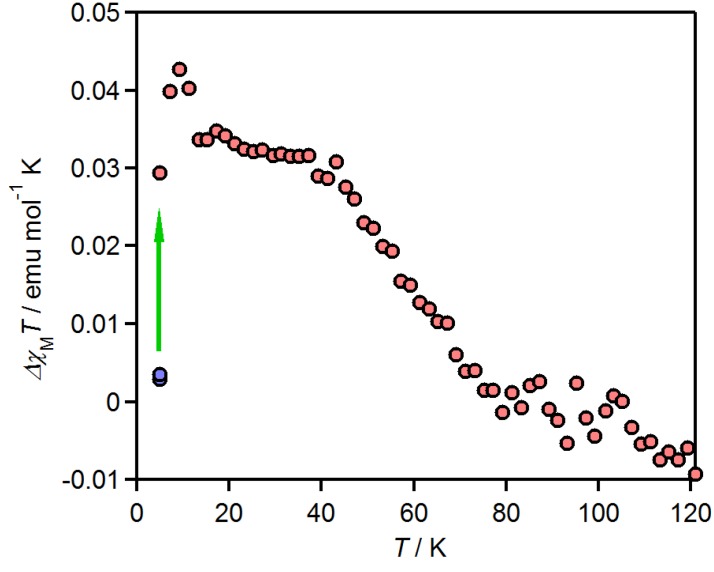
Thermal relaxation of the photo-induced paramagnetism ( Δ *χ*_M_*T*) for [Fe(tdap)_2_(NCS)_2_] [53]. Adapted by permission of The Royal Society of Chemistry.

Light-induced excited spin-state trapping (LIESST) experiments were performed under 0.5 T, in the temperature range of 5–120 K. An Hg-Xe lamp (550 nm, 1.5 mW cm^−2^) was used as a light source. The light, passing through infrared (IR) and green filters, was guided via an optical fiber into the superconducting quantum interference device (SQUID) magnetometer. The sample was placed on the edge of the optical fiber. The thermal relaxation of the paramagnetism induced by photo-irradiation at 5K (Δ*χ*_p_*T*) is shown in Figure 5. Light irradiation brings about a magnetization increase, but is saturated to a small value that is only 1.3% of the theoretical value for a complete LS to HS transition. Since [Fe(tdap)_2_(NCS)_2_] appears as black crystals, only the surface molecules exhibit the spin transition. Upon heating, the photo-induced magnetization disappears at 80 K; this temperature is higher than that of the unsubstituted phenanthroline complex (55 K) [54].

### 2.4. Crystal Structures of tdap Transition Metal Complexes

#### 2.4.1. [M(tdap)_2_X_2_]

**Figure 6:**
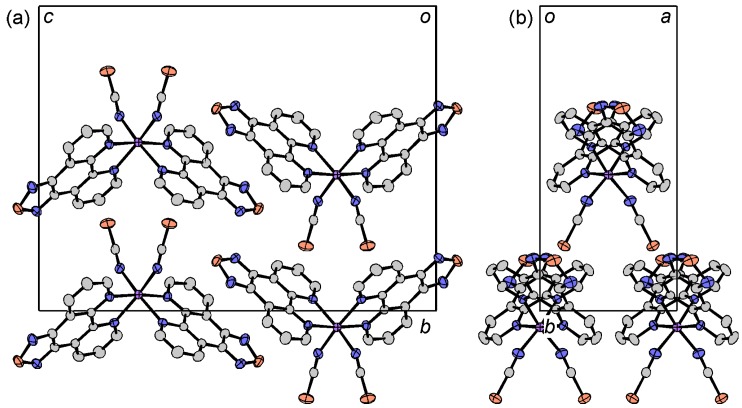
Crystal structure of [Co(tdap)_2_(NCS)_2_].

The crystal structure of the crystal solvent-free cobalt complex [Co(tdap)_2_(NCS)_2_] is shown in Figure 6. The [Co(tdap)_2_(NCS)_2_] complexes are located on a 2-fold rotation axis. The unit cell contains two right-handed and two left-handed enantiomers of this chiral complex. The packing motif of [Co(tdap)_2_(NCS)_2_] is much different from that of the crystal solvent-free iron complex [Fe(tdap)_2_(NCS)_2_] (Figure 4). There are no short intermolecular contacts, although this crystal does not contain crystal solvents which could quench the intermolecular interaction between neighboring complexes as is observed in [Fe(tdap)_2_(NCS)_2_]•MeCN. The molecular arrangement of this crystal seems to be governed only by VDW forces and a small π-interactions (interplanar distances of ~3.47 Å) between tdap moieties.

**Figure 7:**
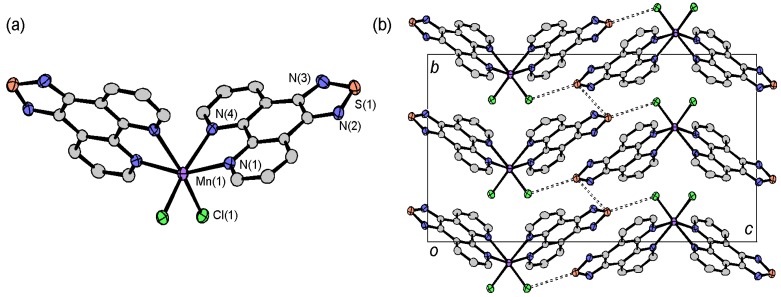
Molecular structure (**a**) and crystal packing (**b**) of [Mn(tdap)_2_Cl_2_].

The molecular structure of [Mn(tdap)_2_Cl_2_] is shown in Figure 7a. The Mn^II^ ion is surrounded by four nitrogen atoms of two tdap ligands, and two chloride ions in *cis* positions. The unit cell contains two right-handed and two left-handed enantiomers of this chiral complex. [Mn(tdap)_2_Cl_2_] complexes are located on a 2-fold rotation axis. The packing of the molecules in the crystals (Figure 7b) reveals S•••S contacts between thiadiazole rings (3.4105(12) Å), and S•••Cl short contacts between a thiadiazole ring and a Cl^−^ ligand (3.5267(11) Å), which are shorter than the sum of VDW radii (3.60 and 3.55 Å, respectively). These short contacts form a two-dimensional (2D) layer.

#### 2.4.2. [M(tdap)_2_X_2_]•solv

In crystals of [M(tdap)_2_(NCS)_2_]•MeCN (M = Mn, Co, Ni, Cu, Zn), the transition metal ions are surrounded by six nitrogen atoms belonging to two isothiocyanate groups in *cis* positions, and two tdap ligands. The unit cell contains two right-handed and two left-handed enantiomers of the chiral complexes. All of these complexes but the Ni complex are crystallized in the *P*2_1_/*n* space group, and the Ni complex is crystallized in the *C*2/*c* space group.

**Figure 8:**
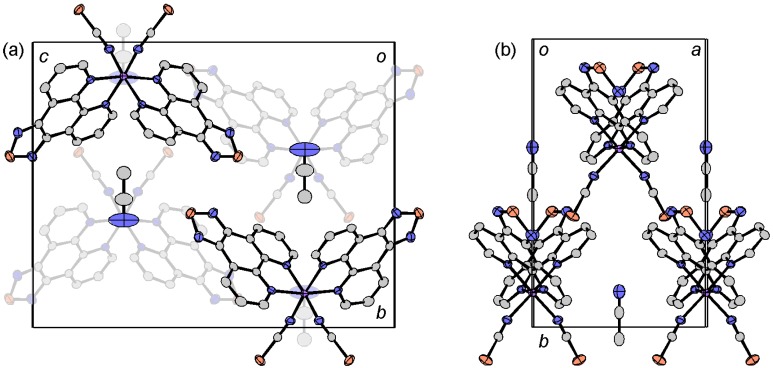
Projections of the unit cell of [Ni(tdap)_2_(NCS)_2_]•MeCN along the *a* (a) and *c* (b) axes.

The crystal structures of [M(tdap)_2_(NCS)_2_]•MeCN (M = Mn, Co, Cu and Zn) are isostructural to that of [Fe(tdap)_2_(NCS)_2_]•MeCN, which is shown Figure 2. Intermolecular contacts between the thiadiazole moiety and acetonitrile are found, and the intermolecular distances found in the Mn, Co, Cu and Zn complexes are 3.074(4), 3.069(4), 3.080(5) and 3.075(3) Å, respectively. The intermolecular interactions between neighboring tdap complexes are longer than that found in the Fe complex, [Fe(tdap)_2_(NCS)_2_]•MeCN at 173 K (Mn: 3.628(7), Co: 3.6251(19), Cu: 3.6333(11) and Zn: 3.6493(6) Å). The crystal structure of [Ni(tdap)_2_(NCS)_2_]•MeCN is shown in Figure 8. In the crystal of the Ni complex, no short intermolecular interaction is found, either between complexes or between a complex and an MeCN molecule.

Magnetic susceptibility measurements of [M(tdap)_2_(NCS)_2_]•MeCN (M = Mn, Co, Ni, Zn) were performed under 0.5 T, in the temperature range of 2–300 K. Curie paramagnetic behavior was found in the Mn, Co and Ni complexes, and the Zn complex exhibited diamagnetism. The measured Curie constants of the Mn, Co, Ni and Zn complexes at 300 K were 4.37, 2.95, 1.22 and 0.0057 emu mol^−1^ K, respectively, which are comparable to (Mn) or higher than (Co, Ni) the theoretical spin-only value. The higher value in the Co and Ni complexes was due to the spin-orbit coupling. Furthermore, the stronger magnetic anisotropy of the Co and Ni ions brought about a gradual decrease of the *χ*_p_*T* value of the Co complex, and a small increase of the *χ*_p_*T* value of the Ni complex.

**Figure 9:**
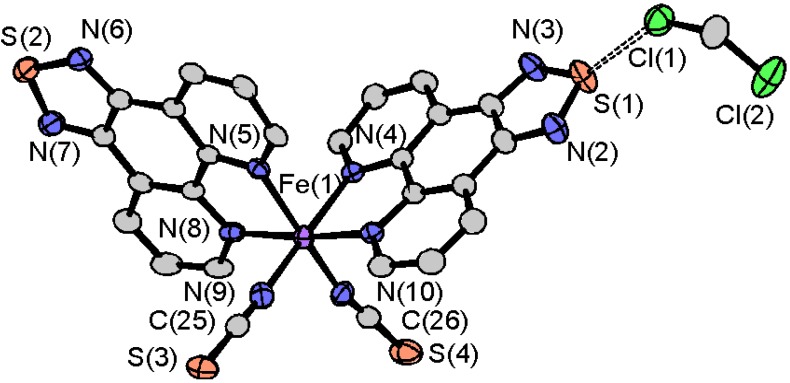
Intermolecular interaction in [Fe(tdap)_2_(NCS)_2_]•CH_2_Cl_2_ [53]. Adapted by permission of The Royal Society of Chemistry.

The molecular structure and intermolecular contact between the complex and crystal solvent for [Fe(tdap)_2_(NCS)_2_]•CH_2_Cl_2_ are shown in Figure 9 [53]. The coordination geometry is nearly the same as those of [Fe(tdap)_2_(NCS)_2_] and [Fe(tdap)_2_(NCS)_2_]•MeCN. The value of the Fe-N bond length is intermediate between the usual HS state and LS state, which implies an SCO transition around this temperature (173 K). The space group and molecular packing of this compound are also nearly the same as for [Fe(tdap)_2_(NCS)_2_]•MeCN (see Figure 2). The S•••Cl contacts are found in this crystal instead of the S•••N contacts found in [Fe(tdap)_2_(NCS)_2_]•MeCN. The magnetic property of [Fe(tdap)_2_(NCS)_2_]•CH_2_Cl_2_ could not be measured due to the low yield.

#### 2.4.3. [Fe(tdap)_3_](NCS)_2_

**Figure 10:**
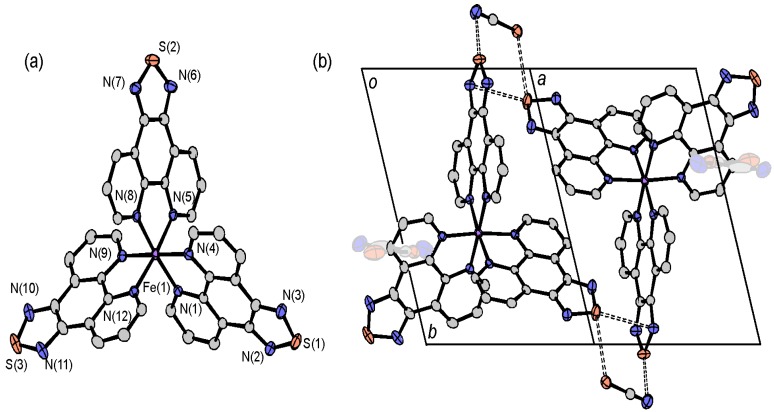
Molecular structure and crystal structure of [Fe(tdap)_3_](NCS)_2_ [53]. Adapted by permission of The Royal Society of Chemistry.

The Fe^II^ ion is surrounded by six nitrogen atoms of three tdap ligands. The unit cell contains two right-handed and two left-handed enantiomers of this chiral complex. The packing of the molecules in the crystals (Figure 10 [53]) reveals S•••N contacts between thiadiazole rings (3.271(2) Å), and S•••N and S•••S short contacts between a thiadiazole ring and a thiocyanate ion (2.994(2) and 3.4597(11) Å), which are shorter than the sum of VDW radii (S•••N: 3.35 Å, S•••S 3.60 Å). These short contacts form a dimer of complexes. There are two crystallographically nonequivalent thiocyanate ions. While one of the thiocyanate ions has a positional disorder, the other ion is fixed on a certain position, maybe due to the intermolecular interaction described above. It appears that the disordered thiocyanate ion simply fills a vacant space.

#### 2.4.4. Dinuclear Copper Complexes

**Figure 11:**
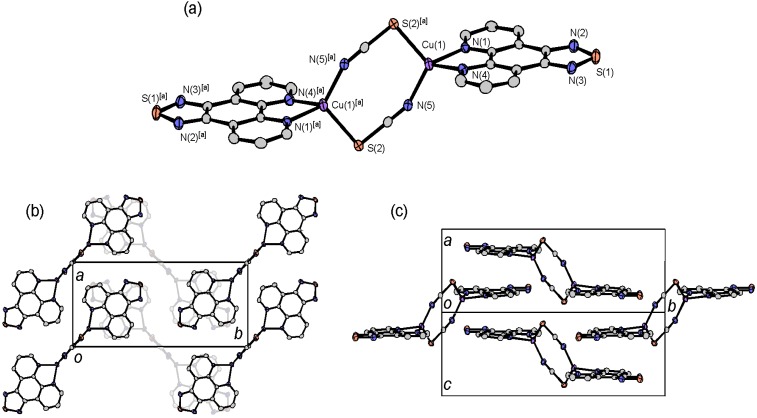
Molecular structure and crystal packing of [Cu_2_(tdap)_2_(NCS)_2_].

The crystal structure of the monovalent copper complex [Cu_2_(tdap)_2_(NCS)_2_] is shown in Figure 11. Two monovalent Cu ions are bridged by two thiocyanate ions. The coordination geometry around Cu ions is distorted tetrahedral, reflecting the *d*^10^ electron configuration of Cu^I^. The tdap moieties form a π stacked column, with an interplanar distance of 3.50 Å, in the direction of the *a* axis. These columns are bridged by the coordination bonds of copper-thiocyanate-copper, and the [Cu_2_(tdap)_2_(NCS)_2_] complexes form a zig-zag sheet structure.

**Figure 12:**
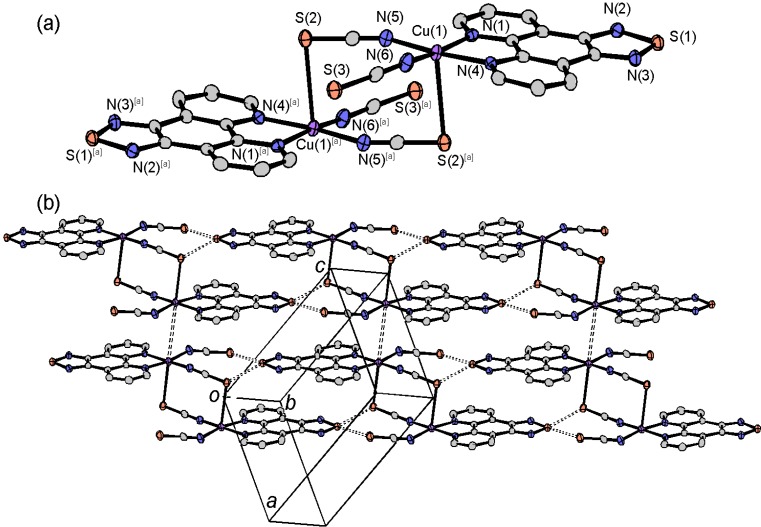
Molecular strucure (**a**) and crystal packing (**b**) of [Cu_2_(tdap)_2_(NCS)_4_].

The crystal structure of the divalent copper complex [Cu_2_(tdap)_2_(NCS)_4_] is shown in Figure 12. Two Cu ions are bridged by two thiocyanate ions. In contrast to [Cu_2_(tdap)_2_(NCS)_2_], an additional thiocyanate ion is coordinated to a Cu ion. The geometry around a Cu^II^ ion is square pyramidal, which is formed by four squarely coordinated nitrogen atoms from a tdap and two thiocyanate ions, and a sulfur atom from a thiocyanate ion on a perpendicular axis to the square. There are short contacts between the sulfur atoms of thiocyanate ions and tdap (3.3632(7) and 3.4073 Å) which form a ladder structure. Two Cu ions face each other across the ladders. The distance between intermolecular Cu ions, which is shown as broken lines in Figure 12, is 3.5812(14) Å.

## 3. Radical Anion Salts of [1,2,5]thiadiazolo[3,4-*f*][1,10]phenanthroline 1,1-dioxide

The molecule tdapO_2_ is designed to possess a stable radical anion species, due to the strong electron-withdrawing property of the thiadiazole dioxide moiety [36,57,58,59], which enhances the acceptor ability of tdapO_2_. In addition, the phenanthroline and thiadiazole dioxide moieties at the molecular ends are expected to operate as a ligand and/or a proton acceptor, which can realize multi-dimensional interactions (Scheme 1b). In this chapter, we describe the electrochemical properties of tdapO_2_ first. Then we describe the multi-dimensional network structures and magnetic properties of the tdapO_2_ radical anion salts, which were reported previously [47,48], and a novel radical anion salt [CoCp_2_]•tdapO_2_.

### 3.1. [1,2,5]thiadiazolo[3,4-f][1,10]phenanthlorine 1,1-dioxide (tdapO_2_)

**Figure 13:**
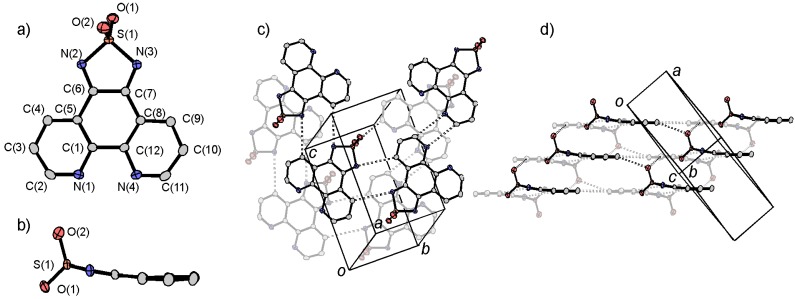
Molecular structure (**a**,**b**) and crystal structure (**c**,**d**) of tdapO_2_. Adapted with permission from [47]. Copyright 2011 American Chemical Society.

The molecule tdapO_2_ was synthesized by a reaction between the diketone precursor (1,10-phenanthroline-5,6-dione [60]) and sulfamide in ethanol [47]. Yellow block crystals of tdapO_2_ can be obtained by sublimation. The molecular structure obtained from the X-ray structure analysis is shown in Figure 13a,b.

The crystal structure of tdapO_2_ consists of a 2D layer formed by C-H•••N hydrogen bonds, as shown by the broken lines in Figure 13c. Figure 13d shows a side view of the layered structure, where the interlayer arrangement includes a S-O•••H hydrogen bond, and very partial π-overlap. Figure 13b suggests the bending of the tdapO_2_ molecule (ca. 15°), which may have been due to the repulsive force between neighboring molecules caused by the out-of-plane S=O bonds.

**Figure 14:**
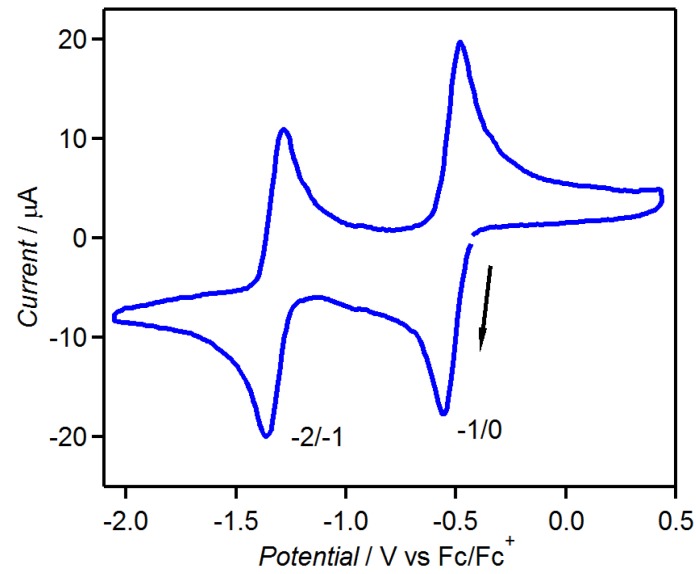
CV of 1 mM tdapO_2_ in MeCN solution of 100 mM TBAClO_4_. Adapted with permission from [47]. Copyright 2011 American Chemical Society.

Figure 14 shows the CV of tdapO_2_, which indicates two reversible reduction peaks at –0.520 and –1.32 V *vs.* Fc^+^/Fc; these processes suggest that the mono and dianionic species of tdapO_2_ are stable and the acceptor ability of tdapO_2_ are comparable to those of the halogen-substituted *p*-benzoquinones [61,62,63,64]. It is notable that the related compound tdap exhibits very weak acceptor ability (see Figure 1). This means that the oxidation of the sulfur in the thiadiazole ring notably reduces the reduction potential.

**Figure 15:**
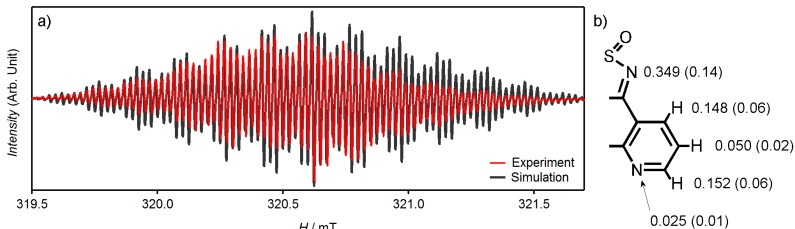
Experimental (red) and simulated (gray) solution EPR spectra of mono-anionic tdapO_2_. The inset shows the hyperfine coupling constants in units of mT used for the simulation, and in parentheses, the estimated spin density on the nitrogen and carbon atoms. Adapted with permission from [48]. Copyright 2011 American Chemical Society.

Solution EPR spectrum measurement of the reduced tdapO_2_ was carried out on a CH_2_Cl_2_ solution of mono-anionic tdapO_2_, which was prepared by the following method. A purple solution of reduced tdapO_2_ in MeCN was prepared by potentiostatic reduction (−0.6 V *vs.* Ag/Ag^+^) of 1 mM tdapO_2_ in an MeCN solution of 100 mM TBAClO_4_. The resulting MeCN solution was then evaporated to dryness and dissolved in CH_2_Cl_2_. This CH_2_Cl_2_ solution was degassed by a frozen degassing technique, and vacuum-sealed in a quartz cell. The measured and simulated EPR spectra are shown in Figure 15. The complex hyperfine structure suggests the existence of an unpaired electron that is well delocalized over the tdapO_2_ skeleton.

Theoretical fitting was carried out, and a best fit was obtained with the following parameters: *g* = 2.0032, two N (*I* = 1) with *a*_N_ = 0.349 mT, two N (*I* = 1) with *a*_N_ = 0.025 mT, two H (*I* = 1/2) with *a*_H_ = 0.152 mT, two H (*I* = 1/2) with *a*_H_ = 0.148 mT, and two H (*I* = 1/2) with *a*_H_ = 0.05 mT, where *I* is the nuclear spin quantum number, and *a*_N_ and *a*_H_ are the hyperfine coupling constants for nitrogen and hydrogen, respectively. The spin distribution on the tdapO_2_ radical anion was estimated from the McConell relation, *a*_H_ = *Q*_C_*ρ*_C_ and *a*_N_ = *Q*_N_*ρ*_N_. *Q*_C_ and *Q*_N_ are the proportionality constants for the hydrogen-neighboring carbon, and for nitrogen, respectively [65,66]. *ρ*_C_ and *ρ*_N_ are the spin distribution on the carbon and nitrogen, respectively. *Q* constants of *Q*_C_ = −2.45 mT [67] for carbon of azaaromatic rings and *Q*_N_ = +2.5 mT [68] for nitrogen were applied. The calculated spin densities are 0.14 and 0.01 for each of the two distinct nitrogen atoms, and 0.06 and 0.02 for the carbon atoms neighboring a hydrogen, at position 2 and 4, and at position 3 (Figure 15 inset), respectively. The positions of each spin density are proposed by comparing the obtained value with the calculated spin density of mono-anionic tdapO_2_ [48].

### 3.2. Synthesis of Intermolecular Compounds and Anion Salts of tdapO_2_

**Figure 16:**
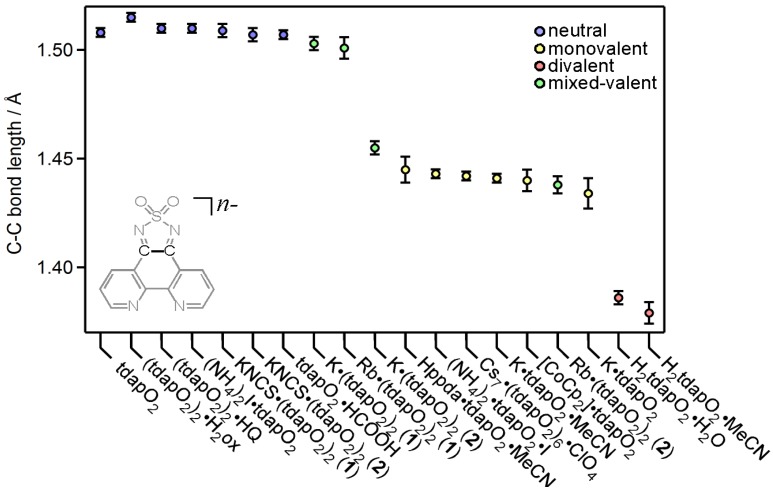
Selected C-C bond lengths of tdapO_2_ compounds for which the structure is known. The colors of the circles represent the stoichiometrically determined valence of the tdapO_2_. Error bars depict ±3 times the standard deviation of the bond lengths.

Radical anion salts of tdapO_2_ were prepared by electrochemical or chemical reduction. KtdapO_2_•0.5MeCN [47,48] and Cs_7_•(tdapO_2_)_6_•ClO_4_ [48] were synthesized by electrochemical reduction of 1 mM solution of tdapO_2_ and saturated KClO_4_ and CsClO_4_ as electrolytes. Higher concentration of tdapO_2_ gave the mixed valent salts K•(tdapO_2_)_2_ [47,48] and Rb•(tdapO_2_)_2_ [48]. Chemical reduction by the slow diffusion method using KI, NH_4_I, *p*-phenylenediamine (ppda) or cobaltocene (Co^II^Cp_2_) as reductants under a N_2_ atmosphere produced K•tdapO_2_ [47,48], (NH_4_)_2_•tdapO_2_•I [48], Hppda•tdapO_2_•MeCN [48] and [Co^III^Cp_2_]•tdapO_2_ [52]. Crystals of intermolecular compounds of neutral tdapO_2_ were also obtained by slow reaction between tdapO_2_, and a proton donor or metal salt, namely formic acid, oxalic acid, hydroquinone, ammonium iodide or potassium thiocyanate, under ambient conditions. Reaction between tdapO_2_ and stronger reductants (ascorbic acid or *N*,*N*,*N*’,*N*’-tetrametyl-1,4-phenylenediamine) gave the divalent tdapO_2_ salts H_2_tdapO_2_•2H_2_O and H_2_tdapO_2_•MeCN, respectively [48]. These intermolecular compounds and salts always exhibited coordination bonding or hydrogen bonding between teapO_2_ and metal ions or proton donors. Single-crystal X-ray analyses for the series of the tdapO_2_ intermolecular compounds and salts revealed a characteristic relation between the bond lengths and the valence of tdapO_2_. Figure 16 shows the selected C-C bond lengths, which are shown in the inset, among the 16 tdapO_2_ compounds. The colors of circles indicate the stoichiometrically determined valence of tdapO_2_ (blue; 0, yellow; −1, red; −2, green; −0.5). The bond lengths between different states are sufficiently distinguishable, and this relationship can be used to determine the valence of tdapO_2_.

### 3.3. Crystal Structures and Magnetic Properties of Radical Anion Salts

#### 3.3.1. Nonsolvated and Solvated Potassium Salts: K•tdapO_2_ and KtdapO_2_•0.5MeCN

**Figure 17:**
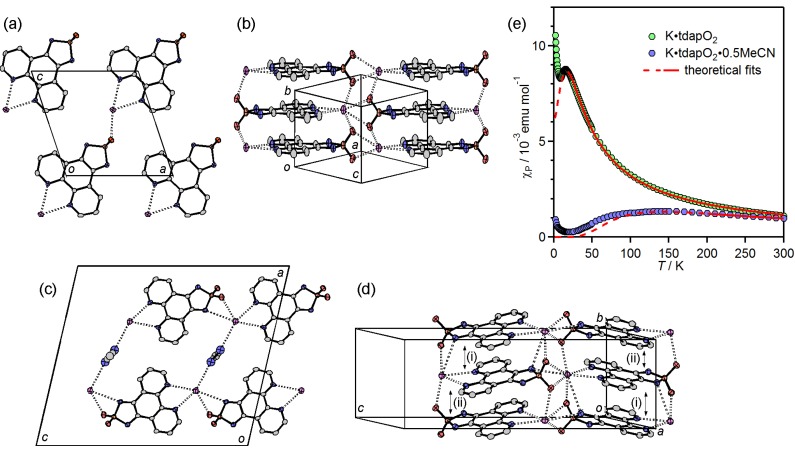
Crystal structure of K•tdapO_2_ (**a**,**b**) and K•tdapO_2_•0.5MeCN (**c**,**d**). Temperature dependence of *χ*_p_ for K•tdapO_2_ and K•tdapO_2_•0.5MeCN (**e**). Adapted with permission from [47] and [48]. Copyright 2011 and 2013 American Chemical Society.

The crystal structure of nonsolvated K•tdapO_2_ is shown in Figure 17a,b. Because the tdapO_2_ molecules are located on a mirror plane, the molecular structure of tdapO_2_ is ideally planar. The crystal structure of K•tdapO_2_ consists of a 2D layer, formed by a coordination polymer chain along the *a* + *c* direction (Figure 17a), and regular π-stacking along the *b* axis with interplanar distances of 3.30 Å (Figure 17b). It thus seems that 2D layers are connected only by VDW force.

**Figure 18:**
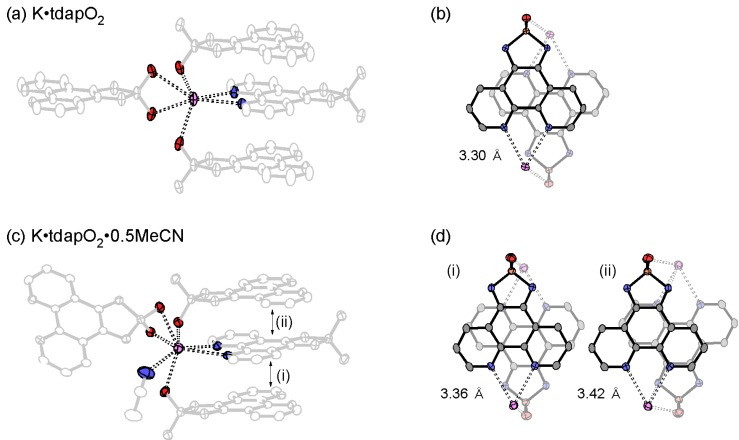
Coordination geometry around the potassium ion (**a**,**c**) and π-overlaps (**b**,**d**) in K•tdapO_2_ (top) and K•tdapO_2_•0.5MeCN (bottom). Adapted with permission from [48]. Copyright 2011 American Chemical Society.

Figure 17c,d show the crystal structure of K•tdapO_2_•0.5MeCN, which consists of a 2D layer formed by π-stacking along the *a* axis and a coordinate bonding chain along the *c* axis (Figure 17c). The 2D layers are connected by coordination bonds of MeCN molecules which are located between two alkali metal ions. The MeCN molecules of K•tdapO_2_•0.5MeCN are disordered between two K ions as shown in Figure 17c. The π-stacking chain includes a very weak alternation with interplanar distances of 3.36 and 3.42 Å. The π-overlaps between tdapO_2_ molecules in these salts (Figure 18b,d) are much larger than that in the neutral tdapO_2_ (Figure 13c), probably due to the exchange interactions between the radical anions.

The coordination geometry around the potassium ion and π-overlaps in K•tdapO_2_ and K•tdapO_2_•0.5MeCN are shown in Figure 18. The π-overlaps between neighboring tdapO_2_ molecules of these two salts are much larger than that of neutral tdapO_2_, probably due to the exchange interaction between radical anions. The π-overlap motifs of the nonsolvated form (Figure 18b) and the solvated form (Figure 18d) are significantly different from each other, presumably due to the geometrical difference around the potassium ions induced by MeCN coordination, as shown in Figure 18a,c.

The measurements of the temperature dependences of the magnetic susceptibility for K•tdapO_2_ and K•tdapO_2_•0.5MeCN were performed under 3 T, in the temperature range of 2–300 K (Figure 17e). The temperature dependences of *χ*_p_ of these radical anion salts were calculated with diamagnetic susceptibilities that were obtained as fitting parameters of the Bonner-Fisher model and Bleaney-Boweres model, respectively.

The *χ*_p_ of K•tdapO_2_ (green circles in Figure 17e) shows a gradual increase with a decrease in temperature from 300 K. After reaching a maximum at 13.5 K, *χ*_p_ shows a decrease, followed by another quick increase below 6.5 K. The latter behavior is probably caused by the Curie spins on the lattice defects. The *χ*_p_ values in the range of 16–300 K can be well fitted to the antiferromagnetic Bonner-Fisher model (Equation (1)), assuming the spin Hamiltonian in the zero field to be *H* = –2*J* Σ*S*_i_•*S*_i+1_, where *J* is the antiferromagnetic coupling constant, and *S*_i_ and *S*_i+1_ are the monoelectronic spin operators [69,70,71]. The numerical expression is

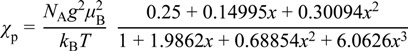
(1)
where *x* = |*J|*/*k*_B_*T*. The solid curve on the open circles in Figure 17e is the theoretical best fit of this model with *g* = 2.00 ± 0.05 and *J*/*k*_B_ = −13 ± 1 K, where *N*_A_ is the Avogadro’s number, *μ*_B_ is the Bohr magneton and *k*_B_ is the Boltzmann constant.

The *χ*_p_ of K•tdapO_2_•0.5MeCN (blue circles in Figure 17e) was smaller than that of K•tdapO_2_, reflecting the alternating nature of the π-stack column. The temperature dependence of *χ*_p_ for K•tdapO_2_•0.5MeCN decreases in temperature from 300 K and forms a broad maximum around 150 K. Further cooling induces a quick increase below 10 K due to Curie defects. The behavior at the high temperature can be fitted to the Bleaney-Bowers model (Equation (2)) with the spin Hamiltonian in zero field: *H* = −2*J* Σ*S*_1_•*S*_2_ [69,72]. The numerical expression is

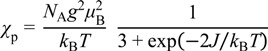
(2)
The *χ*_p_ values in the range of 200–300 K can be well fitted with following fitting parameters: *g* = 2.0 ± 0.1 and *J*/*k*_B_ = −110 ± 10 K.

#### 3.3.2. Mixed Valent Salts: K•(tdapO_2_)_2_ and Rb•(tdapO_2_)_2_

**Figure 19:**
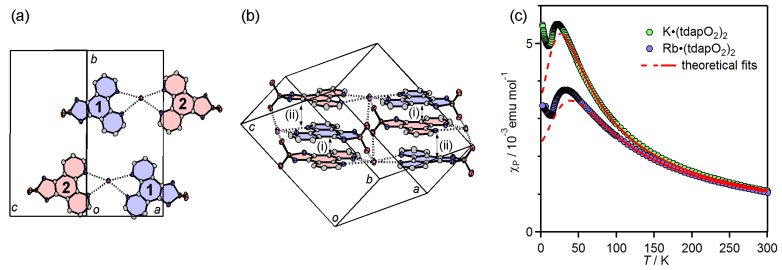
Crystal structure (**a**,**b**) of K•(tdapO_2_)_2_ and temperature dependence of *χ*_p_ (**c**) for K•(tdapO_2_)_2_ and Rb•(tdapO_2_)_2_. Adapted with permission from [47] and [48]. Copyright 2011 and 2013 American Chemical Society.

The crystal structures of K•(tdapO_2_)_2_ and Rb•(tdapO_2_)_2_ were isostructural. Figure 19a,b shows the crystal structure of K•(tdapO_2_)_2_, which consists of two crystallographically nonequivalent tdapO_2_ molecules **1** (blue) and **2** (red). The bond lengths of these two tdapO_2_ molecules (**1** and **2**) are sufficiently distinguishable (green circles in Figure 16), and clearly indicate that one molecule is neutral (**1**) and the other molecule is anionic (**2**). They form an alternating π-stacking arrangement along the *a* + *c* axis, with very similar interplanar distances of 3.29 Å but different intermolecular arrangements (i) and (ii). In a similar way, the tdapO_2_ molecules in Rb•(tdapO_2_)_2_ form an alternating π-stacking arrangement with interplanar distances of 3.29 (i) and 3.31 Å (ii). As shown in Figure 19a, a potassium ion is tweezered by the phenanthroline moieties of the coplanar molecules **1** and **2** in different π-stacking columns, and is capped by the two thiadiazole dioxide moieties from both sides of the plane. These salts are in a class I mixed valence state [73], or, in other words, in a charge-ordered state—a state that has attracted much attention in recent years. Figure 19b indicates that the molecules **1** and **2** exhibit a checker-board-type charge ordering [74].

Figure 19c depicts the temperature dependence of *χ*_p_ for K•(tdapO_2_)_2_ and Rb•(tdapO_2_)_2_, where the molar unit contains two tdapO_2_ molecules. The behavior of K•(tdapO_2_)_2_ (green circles in Figure 19c) in the temperature range from 33 to 350 K can be fitted to the Bonner-Fisher model (Equation (1)) with the following fitting parameters: *g* = 1.95 ± 0.05 and *J*/*k*_B_ = −20 ± 1 K for K•(tdapO_2_)_2_. The behavior of Rb•(tdapO_2_)_2_ (blue circles in Figure 19c) in the temperature range from 54 to 300 K can be fitted to the Bonner-Fisher model (Equation (1)) with the following fitting parameters: *g* = 1.94 ± 0.06 and *J*/*k*_B_ = −29 ± 1 K.

**Figure 20:**
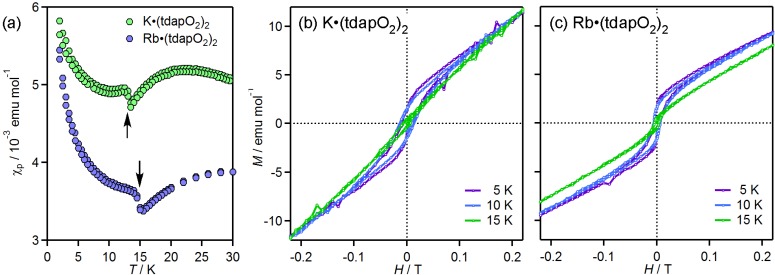
Temperature dependence of *χ*_p_ for K•(tdapO_2_)_2_ and Rb•(tdapO_2_)_2_ at low temperatures (**a**). Magnetization curves of K•(tdapO_2_)_2_ (**b**) and Rb•(tdapO_2_)_2_ (**c**) at 5, 10 and 15 K. Adapted with permission from [47] and [48]. Copyright 2011 and 2013 American Chemical Society.

The |*J*| values of K•(tdapO_2_)_2_ and Rb•(tdapO_2_)_2_ are larger than that of K•tdapO_2_, though the π-stacking column in K•(tdapO_2_)_2_ and Rb•(tdapO_2_)_2_ includes the non-magnetic molecule **1**. In general, the intermolecular magnetic exchange interaction results from cancellation between the ferromagnetic and antiferromagnetic contributions. Therefore, it is not entirely surprising that the antiferromagnetic interactions in K•(tdapO_2_)_2_ and Rb•(tdapO_2_)_2_ are stronger than that in K•tdapO_2_, if the latter involves a stronger ferromagnetic contribution. Below ca. 15K, the *χ*_p_ values of K•(tdapO_2_)_2_ and Rb•(tdapO_2_)_2_ exhibit a sudden jump (Figure 20a), followed by a Curie-like behavior. The field dependence of the magnetization was measured at various temperatures. The magnetization curves (Figure 20b,c) indicate the presence of a small spontaneous magnetization below these temperatures, which is probably caused by spin canting. These are some of the highest transition temperatures among molecule-based magnetic materials that do not include transition metals [18].

#### 3.3.3. Kagome Lattice Formed by Coordination Bonding: Cs_7_•(tdapO_2_)_6_•ClO_4_

**Figure 21:**
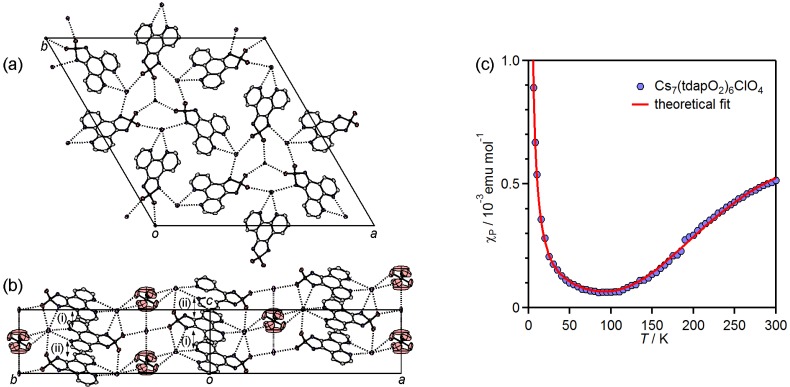
Crystal structure (**a**,**b**) and temperature dependence of *χ*_p_ (**c**) for Cs_7_•(tdapO_2_)_6_•ClO_4_. Adapted with permission from [48]. Copyright 2013 American Chemical Society.

Figure 21a,b show the crystal structure of Cs_7_•(tdapO_2_)_6_•ClO_4_, which consists of seven crystallographically nonequivalent cesium ions (one is on the 3-fold rotation axis and inversion center, and the others are in the general positions), one perchlorate ion on the 3-fold rotation axis and inversion center, and six crystallographically equivalent tdapO_2_ molecules. The positions of oxygen atoms belonging to a ClO_4_ ion are disordered and their occupancies are fixed to 0.5. This stoichiometric result and the bond lengths of tdapO_2_ (Figure 16) suggest that the valence of tdapO_2_ is −1 and the composition formula is Cs_7_•(tdapO_2_)_6_•ClO_4_. A Cs ion is surrounded by five oxygen atoms belonging to five tdapO_2_ and four nitrogen atoms belonging to three tdapO_2_. The other Cs ion is surrounded by six oxygen atoms belonging to six tdapO_2_ and eight oxygen atoms (whose occupancy is 0.5) belonging to two ClO_4_ ions. A notable point is that six tdapO_2_ radical anions are located on a so-called “Kagomé lattice,” in which antiferromagnetically interacted spins form a frustrated system [75]. They also form a strongly alternating π-stacking arrangement along the *c* axis, with interplanar distances of 3.13 (i) and 3.29 Å (ii).

Figure 21c depicts the temperature dependence of *χ*_p_ for Cs_7_•(tdapO_2_)_6_•ClO_4_, where the molar unit contains one tdapO_2_ molecule. With the decrease in temperature from 300 K, *χ*_p_ shows a gradual decrease. After reaching a minimum around 100 K, the value of *χ*_p_ is maintained down to 30 K, and then shows another quick increase. The latter behavior is probably caused by the Curie spins on the lattice defects, whose Curie constant is calculated as 0.0037 emu mol^−1^ K. The behavior of Cs_7_•(tdapO_2_)_6_•ClO_4_ in the temperature range from 30 to 300 K can be fitted to the Bleaney-Bowers model. The best fit was obtained with the following fitting parameters: *g* = 2.0013 (fixed) and *J*/*k*_B_ = −310 ± 10 K for Cs_7_•(tdapO_2_)_6_•ClO_4_. The *g* value was fixed at 2.0013, which was obtained from solid EPR measurement for a microcrystalline sample of Cs_7_•(tdapO_2_)_6_•ClO_4_.

#### 3.3.4. Ammonium Salts: (NH_4_)_2_•tdapO_2_•I and Hppda•tdapO_2_•MeCN

**Figure 22:**
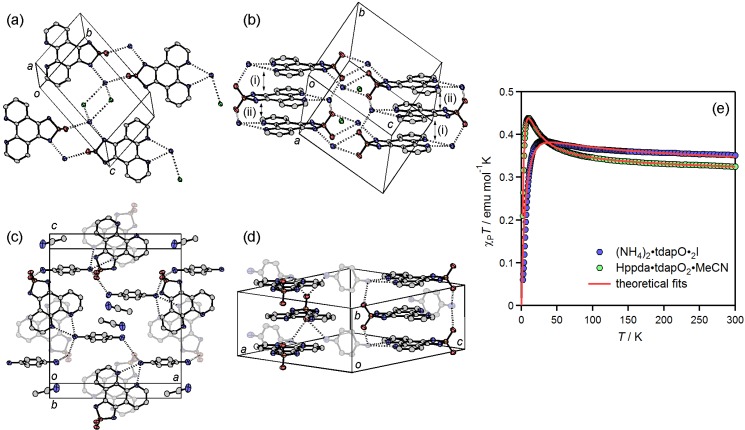
Crystal structure of (NH_4_)_2_•tdapO_2_•I (**a**,**b**) and Hppda•tdapO_2_•MeCN (**c**,**d**). Temperature dependence of *χ*_p_*T* for (NH_4_)_2_•tdapO_2_•I and Hppda•tdapO_2_•MeCN (**e**). Adapted with permission from [48]. Copyright 2013 American Chemical Society.

**Figure 23:**
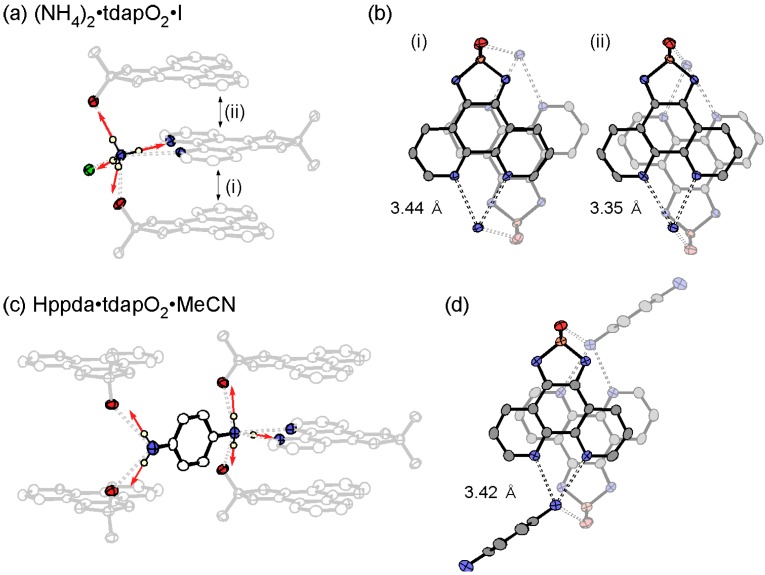
Hydrogen-bonding geometry around ammonium ions (**a**,**c**) and π-overlaps (**b**,**d**) of (NH_4_)_2_•tdapO_2_•I (top) and Hppda•tdapO_2_•MeCN (bottom). Adapted with permission from [48]. Copyright 2013 American Chemical Society.

Figure 22a,b shows the crystal structure of (NH_4_)_2_•tdapO_2_•I, which consists of an alternating π stacking of tdapO_2_ with interplanar distances of (i) 3.35 and (ii) 3.44 Å (see Figure 22b). These are longer than those observed in the alkali metal salts. The intermolecular π-overlaps, (i) and (ii), in the stacking chain are shown in Figure 23b. Both the phenanthroline and thiadiazole dioxide moieties form hydrogen bonds to different ammonium ions, indicating the proton-accepting ability of the tdapO_2_ radical anion (Figure 23a). Between the stacking columns, a 2D hydrogen-bonding network is formed by NH_4_^+^, I^−^ and the proton-accepting moieties of tdapO_2_.

The crystal structure of Hppda•tdapO_2_•MeCN is shown in Figure 22c,d. Because it is difficult to determine the existence of the protons by XRD analysis, due to their small electron density, the valence of tdapO_2_ was confirmed to be monovalent from the bond lengths of the crystal structure (Figure 16). This result implies that a ppda molecule is protonated and becomes a 4-aminoanilinium ion (Hppda). The geometry of the hydrogen bonds around N(5), shown in Figure 23c, also implies a protonation of N(5). The crystal structure of Hppda•tdapO_2_•MeCN consists of a regular π-stacking of tdapO_2_ with interplanar distances of 3.42 Å (Figure 22d). This distance is comparable to those of (NH_4_)_2_•tdapO_2_•I. The intermolecular π-overlap in the stacking chain is shown in Figure 23d. The π-stack columns are connected by hydrogen bondings between tdap radical anions and Hppda cations, and form a 3D hydrogen-bonding network structure.

The blue circles in Figure 22e show the temperature dependence of *χ*_p_*T* for (NH_4_)_2_•tdapO_2_•I. When the temperature decreases from 300 K, *χ*_p_*T* exhibits a gradual increase down to 30 K. After passing a broad maximum, *χ*_p_*T* decreases quickly. This behavior indicates the coexistence of ferromagnetic and antiferromagnetic interactions, with the former being stronger than the latter. Since the crystal structure consists of alternated π-stacking, this temperature dependence can be interpreted in terms of a ferromagnetic dimer, with weak antiferromagnetic interdimer coupling (*H* = −2*J*_1_*S*_1_•*S*_2_ − 2*zJ*_2_(⟨*S*_1_^z^⟩•*S*_2_^z^ + ⟨*S*_2_^z^⟩•*S*_1_^z^) [76] using

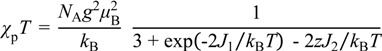
(3)
where *J*_1_ and *J*_2_ express the intradimer ferromagnetic coupling constant, and the interdimer antiferromagnetic coupling constant, respectively, and *z* is the number of the nearest neighbors connected by *J*_2_. The solid curve on blue circles in Figure 22e is the theoretical best fit with *g* = 1.94 ± 0.10, *J*_1_/*k*_B_ = 24 ± 1 K and *zJ*_2_/*k*_B_ = −7.9 ± 0.1 K. It is clear that the tdapO_2_ radical anion has the potential to yield both ferromagnetic and antiferromagnetic intermolecular interactions.

The green circles in Figure 22e show the temperature dependence of *χ*_p_*T* for Hppda•tdapO_2_•MeCN. When the temperature decreases from 300 K, *χ*_p_*T* exhibits a gradual increase down to 10 K. After passing a broad maximum, *χ*_p_*T* decreases quickly. This behavior is similar to that of (NH_4_)•tdapO_2_•I. Since the crystal structure consists of regular π-stacking, this temperature dependence can be interpreted in terms of a ferromagnetic 1D chain with weak antiferromagnetic interchain coupling (*H* = −2*J*_1_Σ*S*_i_•*S*_i+1_ and a molecular mean-field correction) [69,71,77] using

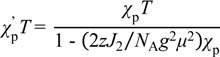
(4)
where *χ*_p_ = (*N*_A_*g*^2^*μ*_B_^2^)/(4*k*_B_*T*)[*N*/*D*]^2⁄3^, *N* = 1.0 + 5.7979916*x* + 16.902653*x*^2^ + 29.376885*x*^3^ + 29.832959*x*^4^ + 14.036918*x*^5^, *D* = 1.0 + 2.7979916*x* + 7.0086780*x*^2^ + 8.6538644*x*^3^ + 4.5743114*x*^4^, and *x* = *J*_1_⁄(*k*_B_*T*). The *χ*’_p_ and *χ*_p_ are the magnetic susceptibility with and without mean-field correction, respectively. The parameters *J*_1_ and *J*_2_ express the intrachain ferromagnetic coupling constant and the interchain antiferromagnetic coupling constant, respectively, and *z* is the number of the nearest neighbors connected by *J*_2_. The solid curve on green circles in Figure 22e is the theoretical best fit with *g* = 1.84 ± 0.10, *J*_1_/*k*_B_= 5.6 ± 0.1 K and *zJ*_2_/*k*_B_= −2.2 ± 0.1 K. This small *g* value indicates the possible decomposition of tdapO_2_ radical anions, lattice defect of the protons or diamagnetic impurities such as incompletely dried MeCN solvent.

#### 3.3.5. Cobaltocenium Salt: [CoCp_2_]•tdapO_2_

**Figure 24:**
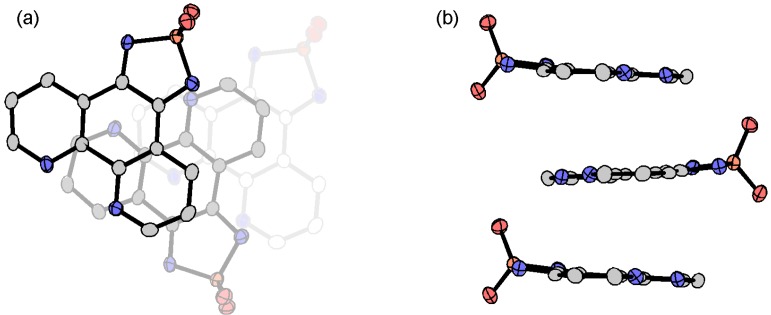
Molecular arrangement of [CoCp_2_]•tdapO_2_.

Crystals of novel radical anion salt [CoCp_2_]•tdapO_2_ were obtained by diffusion method using cobaltocene and tdapO_2_ in CH_2_Cl_2_. Crystallographic parameters for [CoCp_2_]•tdapO_2_ are shown in Table S1 (Supplementary Materials). The valence of tdapO_2_ could be determined as −1 from its bond length (Figure 16). The π-overlap arrangements of [CoCp_2_]•tdapO_2_ are shown in Figure 24. The intermolecular arrangement of the tdapO_2_ radical anions in this system is much different from those in the other radical anion salts. The molecular planes of tdapO_2_ are not parallel. The overlapped part shows a closer interplanar distance, maybe due to the exchange interaction through π orbitals.

**Figure 25:**
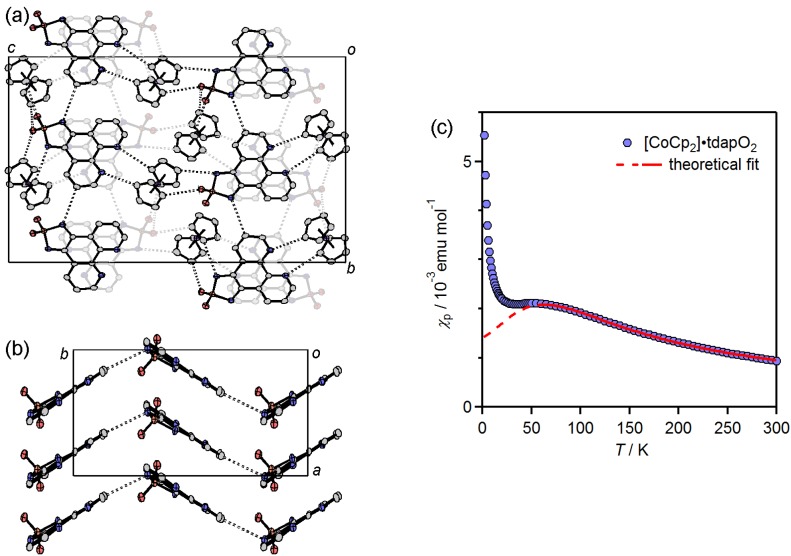
Crystal structure (**a**,**b**) and temperature dependence of *χ*_p_ (**c**) for CoCp_2_•tdapO_2_.

Figure 25a,b shows the crystal structure of [CoCp_2_]•tdapO_2_. The tdapO_2_ molecules form a 2D layer consisting of regular π stacking and weak C-H•••N hydrogen bonds. These layers are connected by weak C-H•••N and C-H•••O hydrogen bonds via cobaltocenium ions. Cobaltocenium ions have neither vacant coordinate sites nor strong proton-donor ability, and are the sole example of a salt lacking both attributes among the obtained tdapO_2_ salts.

The temperature dependence of *χ*_p_ for [CoCp_2_]•tdapO_2_ is shown in Figure 25c. With a decrease in temperature from 300 K, *χ*_p_ shows a gradual increase. After reaching a maximum around 50 K, *χ*_p_ shows a slight decrease, followed by another quick increase below 20 K. The latter behavior is probably caused by the Curie spins on the lattice defects. The structural study revealed that the tdapO_2_ π-stack column is well separated by the diamagnetic cation [CoCp_2_]^+^. The *χ*_p_ values in the range of 60–300 K can be well fitted to the antiferromagnetic Bonner-Fisher model (Equation (1)). The solid curve on the circles in Figure 25c is the theoretical best fit of this model with *g* = 1.92 ± 0.10 and *J*/*k*_B_ = −49 ± 5 K.

### 3.4. Theoretical Calculations

**Figure 26:**
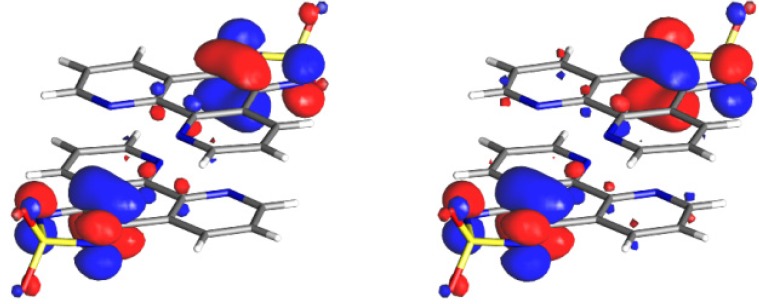
Active MOs resulting from a CAS[2,2]SCF calculation performed for the triplet state of a dimer extracted from the crystal structure of K•tdapO_2_. Adapted with permission from [48]. Copyright 2013 American Chemical Society.

It is quite remarkable that the tdapO_2_ radical anion salts exhibit exchange-coupling constants that vary significantly from antiferromagnetic *J*/*k*_B_ = −310 K to ferromagnetic *J*/*k*_B_ = 24 K values (see Table 2). Wave function-based ab initio difference-dedicated configuration interaction (DDCI) calculations for the exchange interactions of 6 different radical dimers that were found in four kinds of tdapO_2_ salts were carried out [48]. The exchange coupling constants *J*, effective hopping integral *t*, on-site repulsion *U*, and direct exchange *K* are summarized in Table 2. The ferro- and antiferromagnetic contributions can be estimated by following the 2*J* = 2*K* − 4*t*^2^/*U* partitioning, with the 2*K* term representing the ferromagnetic contribution and the −4*t*^2^/*U* term the antiferromagnetic contribution. The *U* values for tdapO_2_ salts are almost constant, in agreement with the finding of Rota *et al.* that the *U* values are mostly governed by building units [78]. The magnetic orbitals are the in-phase and out-of-phase linear combinations of the singly occupied MOs (SOMOs) localized on each radical (Figure 26). The relative position of the radicals *(i.e*., the three-dimensional arrangement) becomes a determining factor governing the magnetic interactions reflecting the localized character of the spin densities. Table 2 shows the evolution of the *J* value with respect to the interplanar distance between the radical dimers. The significant dependence of the exchange coupling constant on this structural parameter is very instructive and indicates that the overlap between the monomers plays a major role in defining the magnetic character of these materials. The calculated exchange coupling constant values are in good agreement with the experimental estimates (Table 2), despite some qualitative deviations observed in weakly interacting systems. 

The comparison between Hppda•tdapO_2_•MeCN and the hypothetical Hppda•tdap•MeCN (lacking oxygen atoms on the thiadiazole ring) is rather instructive. Removal of the oxygen atoms shifts the magnetic behavior from ferro- (13 K) to antiferromagnetic (−4 K). It is remarkable that removal of the electron-withdrawing oxygen atoms leads to a ca. 3800 K increase of *U* in the hypothetical Hppda•tdap•MeCN radical as compared to Hppda•tdapO_2_•MeCN. Such variation of *U* is overtaken by a significant increase of the hopping integral *t* (from 85 to 791 K), which drives the hypothetical Hppda•tdap•MeCN into the antiferromagnetic regime. The presence of oxygen atoms reduces the superexchange contribution (absolute value of the antiferromagnetic contribution) with competitive effects on the parameters *t* and *U*.

**Table 2:** Interplanar distances (*d*_int_) and magnetic exchange coupling constants (*J*_1_ and *J*_2_) from the theoretical fits of experimental data, and *J*_calc_ and corresponding *t*, *K*, and *U* parameters from DDCI calculations performed on radical anion pairs. Adapted with permission from [48]. Copyright 2013 American Chemical Society.

		Experimental data		Computational calculation			
		*d*_int_/Å	*J*_1_*k*_B_^−1^/K (*zJ*_2_*k*_B_^−1^/K)	*J*_calc_*k*_B_^−1^/K	*tk*_B_^−1^/K	*Kk*_B_^−1^/K	*Uk*_B_^−1^/K
K•tdapO_2_		3.30	−13	17	72	23	30522
K•tdapO_2_•0.5MeCN	(i)	3.36	−110	−100	1352	26	29355
	(ii)	3.42		8	237	13	30118
K•(tdapO_2_)_2_	(i)	3.29	−20				
	(ii)	3.29					
Rb•(tdapO_2_)_2_	(i)	3.29	−29				
	(ii)	3.31					
Cs_7_•(tdapO_2_)_6_•ClO_4_	(i)	3.13	−310				
	(ii)	3.36					
(NH_4_)_2_•tdapO_2_•I	(i)	3.44	24	13	142	17	30709
	(ii)	3.35	(−7.9)	6	208	13	30876
Hppda•tdapO_2_•MeCN		3.42	5.6	13	85	17	30745
			(−2.2)				
Hppda•tdap•MeCN		Hypothetical system		−4	791	32	34533
[CoCp_2_]•tdapO_2_			−49				

## 4. Summary

The 1,2,5-thiadiazole compound tdap was induced to transition metal complexes in an effort to enhance intermolecular interactions and form highly networked systems. As a result, some of these complexes exhibited S•••S, S•••N, or S•••X intermolecular interactions, and formed a dimer, 1D ladder, 2D layer, and three-dimensional (3D) network structure. These interactions could be classified into four types: S(tdap)•••S(tdap), S(tdap)•••X (X = S, Cl belonging to coordinated anion), S(tdap)•••N(tdap), and S(tdap)•••X (X = N, Cl, S belonging to packing molecule or anion). The packing molecules and anions tended to quench the network structure in transition metal complexes of tdap. The effect of the intermolecular interaction on the physical properties was shown in the spin-crossover complex by the shift in their transition temperatures. [Fe(tdap)_2_(NCS)_2_]•MeCN and [Fe(tdap)_2_(NCS)_2_], underwent an SCO transition whose critical temperatures (*T*_c_) were 210 and 315 K, respectively. A significant shift of transition temperature was found in solvent-free [Fe(tdap)_2_(NCS)_2_], and may have been due to the chemical pressure caused by strong intermolecular interactions.

The 1,2,5-thiadiazole dioxide compound tdapO_2_ was designed as a stable electron acceptor molecule with coordination sites. The electrochemistry of this compound indicated its good acceptor ability. The chemical and electrochemical reduction successfully gave 8 kinds of radical anion salts. These salts formed highly networked structures governed by effective coordination or hydrogen bonds. Most of the radical anion salts exhibited 1D magnetic behavior with various magnetic coupling constants that ranged from strong antiferromagnetic (−320 K) to ferromagnetic (24 K). Mixed-valent salts K•(tdapO_2_)_2_ and Rb•(tdapO_2_)_2_ showed magnetic ordering at low temperature, indicating the existence of considerable intercolumnar magnetic interactions in these salts.

Whereas the intermolecular interaction and physical properties of 1,2,5-thiadiazole compounds have been extensively reported on, for many systems, the solid state physical properties of 1,2,5-thiadiazolo 1,1-dioxide compounds have just started to be investigated. As shown in this review, 1,2,5-thiadiazole 1,1-dioxide compounds exhibit unique properties, such as coordination ability, proton acceptor ability, electron acceptor ability, and stability of radical anion species, with properties very different from those of 1,2,5-thiadiazole compounds. We expect the 1,2,5-thiadiazole 1,1-dioxide unit to form a new class of compounds which show exotic properties similar to those achieved for other heterocyclic chalcogen-nitrogen compound, such as thiadiazole, dithiazole, dithiadiazole *etc.*

## Supplementary Materials

Supplementary materials can be accessed at: http://www.mdpi.com/1420-3049/19/1/609/s1.
