# Supplementary material for: Transition Metal Complexes and Radical Anion Salts of 1,10-Phenanthroline Derivatives Annulated with a 1,2,5-Tiadiazole and 1,2,5-Tiadiazole 1,1-Dioxide Moiety: Multidimensional Crystal Structures and Various Magnetic Properties
Source: Molecules. 2014 Jan 7;19(1):609–40. doi: 10.3390/molecules19010609 (PMC6271449; doi:10.3390/molecules19010609)
Supplement: Supplementary file 1 [file molecules-19-00609-s001.pdf]

Supplementary Materials for Transition Metal Complexes and Radical Anion Salts of 1,10-Phenanthroline Derivatives Annulated with a 1,2,5-Tiadiazole and 1,2,5-Tiadiazole 1,1-Dioxide Moiety: Multidimensional Crystal Structures and Various Magnetic Properties

Experimental Details

X-ray Crystal Structural Analyses

X-ray investigations were performed at 320 K and 120 K for $[Fe(tdap)_2(NCS)_2]$•MeCN and at 173 K for the other compounds. Crystals were mounted on a loop using oil (CryoLoop, Immersion Oil, Type B; Hampton Research Corp. Aliso Viejo, CA, USA) for low temperature measurements and on a quartz fiber using epoxy bond (Araldite Rapid; Huntsman Corp, The Woodlands, TX, USA) and set on a Rigaku RA-Micro007 with a Saturn CCD detector by using graphite-monochromated Mo Kα radiation (λ = 0.710690 Å) under a nitrogen stream. The frame data were integrated and corrected for absorption with the Rigaku/MSC CrystalClear package. The structures were solved by direct methods and standard difference map techniques, and were refined with full-matrix least-square procedures on F^2 by using the Rigaku/MSC CrystalStructure package. Anisotropic refinement was applied to all non-hydrogen atoms. Hydrogen atoms in cobaltocenium ion of $[CoCp_2]$•$tdapO_2$ were located in a difference Fourier map and refined isotropically. All other hydrogen atoms were placed at the calculated positions and refined using a riding model. Crystallographic parameters are shown in Table S1.

Magnetic Measurements

Magnetic susceptibility measurements for $[M(tdap)_2(NCS)_2]$•MeCN (M = Mn, Fe, Co, Ni, Zn) and $[CoCp_2]$•$tdapO_2$ were carried out on polycrystalline samples on a MPMS-SL Quantum Design magnetometer. A quartz glass tube for $[Fe(tdap)_2(NCS)_2]$•MeCN and a plastic straw for the other compounds were used as the sample folder. Measurements were performed under 0.1 T for $[M(tdap)_2(NCS)_2]$•MeCN (M = Mn, Fe, Co, Ni, Zn) and under 3T for $[CoCp_2]$•$tdapO_2$. The temperature dependences of the paramagnetic susceptibilities χ_p were calculated from theoretical fitting using the diamagnetic susceptibility as a fitting parameter.

Table S1. Crystallographic parameters.

	$[Fe(tdap)_2(NCS)_2]$•MeCN		$[Co(tdap)_2(NCS)_2]$	$[Mn(tdap)_2(NCS)_2]$ •MeCN
	HS state	LS state		
Formula	$C_{28}H_{15}N_{11}S_4Fe$		$C_{26}H_{12}N_{10}CoS_4$	$C_{28}H_{15}N_{11}S_4Mn$
Formula weight	689.59		651.62	688.68
Dimension/mm^3	0.18 × 0.15 × 0.09		0.10 × 0.05 × 0.01	0.15 × 0.07 × 0.06
T/K	320	120	173	173
Crystal system	Monoclinic	Monoclinic	Monoclinic	Monoclinic
Space group	$P2_1/n$ (#14)	$P2_1/n$ (#14)	$C2/c$ (#15)	$P2_1/n$ (#14)
a/Å	11.7576(14)	11.525(2)	7.476(2)	11.7681(17)
b/Å	18.829(2)	18.670(3)	16.514(5)	18.664(3)
c/Å	13.3450(15)	12.992(2)	21.678(6)	13.3313(18)
α /°				
β /°	99.4530(15)	98.722(2)	96.728(4)	100.218(2)
γ /°				
V/Å^3	2914.3(6)	2763.2(8)	2657.8(13)	2881.7(7)
Z	4	4	4	4
D_{calc}/g cm^{-3}	1.572	1.658	1.628	1.587
μ(Mo Kα)/cm^{-1}	8.456	8.919	9.996	7.903
F(000)	1400.00	1400.00	1316.00	1396.00
$2\theta_{max}$/°	54.9	54.9	55.0	55.0
Reflections collected	22443	21585	10575	22973
Unique reflections (R_{int})	6544	6206	2980	6556
	(0.025)	(0.028)	(0.050)	(0.045)
Number of parameters	398	398	187	398
Final $R_1[I > 2\sigma(I)]$ [a]	0.0366	0.0378	0.0474	0.0411
wR_2 [b]	0.0958	0.0912	0.1172	0.1082
Goodness-of-fit	1.044	1.040	1.067	1.078

Table S1. *Cont.*

	**[Co(tdap)$_2$(NCS)$_2$] •MeCN**	**[Ni(tdap)$_2$(NCS)$_2$] •MeCN**	**[Cu(tdap)$_2$(NCS)$_2$] •MeCN**	**[Zndap)$_2$(NCS)$_2$] •MeCN**
Formula	$C_{28}H_{15}N_{11}S_4Co$	$C_{28}H_{15}N_{11}S_4Ni$	$C_{28}H_{15}N_{11}S_4Cu$	$C_{28}H_{15}N_{11}S_4Zn$
Formula weight	692.67	692.44	697.29	699.12
Dimension/mm^3	0.12 × 0.09 × 0.08	0.07 × 0.07 × 0.04	0.20 × 0.20 × 0.20	0.15 × 0.10 × 0.10
T/K	173	173	173	173
Crystal system	Monoclinic	Monoclinic	Monoclinic	Monoclinic
Space group	$P2_1/n$ (#14)	$C2/c$ (#15)	$P2_1/n$ (#14)	$P2_1/n$ (#14)
a/Å	11.6350(18)	9.491(2)	11.6129(19)	11.6761(11)
b/Å	18.691(3)	15.380(4)	18.761(3)	18.7392(17)
c/Å	13.203(2)	19.967(5)	13.179(2)	13.2026(12)
α /°				
β /°	99.627(3)	102.708(4)	98.535(2)	99.4276(13)
γ /°				
V/Å^3	2830.8(8)	2843.2(12)	2839.5(8)	2849.7(5)
Z	4	4	4	4
D_{calc}/g cm^{-3}	1.625	1.617	1.631	1.629
μ(Mo Kα)/cm^{-1}	9.448	10.193	11.066	11.982
F(000)	1404.00	1408.00	1412.00	1416.00
$2\theta_{max}$/°	55.0	55.0	55.0	55.0
Reflections collected	22649	11326	22632	22576
Unique reflections (R_{int})	6409 (0.043)	3192 (0.041)	6405 (0.039)	6487 (0.026)
Number of parameters	398	202	398	398
Final $R_1[I > 2\sigma(I)]$ [a]	0.0438	0.0394	0.0471	0.0298
wR_2 [b]	0.1126	0.0890	0.1074	0.0711
Goodness-of-fit	1.062	1.072	1.089	1.049

Table S1. *Cont.*

	**[Mn(tdap)$_2$Cl$_2$]**	**[Cu$_2$(tdap)$_2$(NCS)$_2$]**	**[Cu$_2$(tdap)$_2$(NCS)$_4$]**	**[CoCp$_2$]•tdapO$_2$**
Formula	$C_{24}H_{12}N_8S_2Cl_2Mn$	$C_{26}H_{12}N_{10}S_4Cu_2$	$C_{14}H_6N_6S_3Cu$	$C_{22}H_{16}N_4O_2SCo$
Formula weight	602.38	719.78	417.97	459.39
Dimension/mm^3	0.20 × 0.20 × 0.01	0.15 × 0.05 × 0.01	0.10 × 0.05 × 0.02	0.10 × 0.07 × 0.01
T/K	173	173	173	173
Crystal system	Monoclinic	Monoclinic	Triclinic	Orthorhombic
Space group	$C2/c$ (#15)	$P2_1/c$ (#14)	$P\bar{1}$ (#2)	$Pbca$ (#61)
a/Å	8.277(3)	9.1233(17)	8.2240(19)	8.274(4)
b/Å	12.142(5)	19.608(4)	8.314(2)	16.528(7)
c/Å	22.935(9)	7.5173(14)	11.979(3)	27.116(12)
α/°			102.996(3)	
β/°	93.665(6)	109.386(2)	105.900(4)	
γ/°			90.120(3)	
V/Å^3	2300.1(16)	1268.5(4)	765.7(3)	3708(3)
Z	4	2	2	8
D_{calc}/g cm^{-3}	1.739	1.884	1.813	1.646
μ(Mo Kα)/cm^{-1}	10.221	20.482	18.436	10.679
F(000)	1212.00	720.00	418.00	1880.00
$2\theta_{max}$/°	54.9	55.0	55.0	55.0
Reflections collected	8983	9991	6169	26180
Unique reflections (R_{int})	2618 (0.071)	2810 (0.062)	3376 (0.023)	4254 (0.065)
Number of parameters	169	191	218	312
Final $R_1[I > 2\sigma(I)]$ [a]	0.0519	0.0309	0.0307	0.0683
wR_2 [b]	0.1150	0.0813	0.0741	0.1385
Goodness-of-fit	1.131	0.859	1.063	1.141

[a] $R_1 = \Sigma||F_o|-|F_c||/\Sigma|F_o|$; [b] $wR_2 = [\Sigma\{w(F_o^2-F_c^2)^2\}/\Sigma w(F_o^2)^2]^{1/2}$.
